# Sporotrichosis Cluster in Domestic Cats and Veterinary Technician, Kansas, USA, 2022

**DOI:** 10.3201/eid3005.231563

**Published:** 2024-05

**Authors:** Ian Hennessee, Erin Barber, Erin Petro, Stephanie Lindemann, Bryan Buss, Amanda Santos, Lalitha Gade, Shawn R. Lockhart, D. Joseph Sexton, Tom Chiller, Mitsuru Toda

**Affiliations:** Centers for Disease Control and Prevention, Atlanta, Georgia, USA (I. Hennessee, B. Buss, A. Santos, L. Gade, S.R. Lockhart, J. Sexton, T. Chiller, M. Toda);; Republican Valley Veterinary Clinic, Saint Francis, Kansas, USA (E. Barber);; Kansas Department of Health and Environment, Topeka, Kansas, USA (E. Petro, S. Lindemann);; Nebraska Department of Health and Human Services, Lincoln, Nebraska, USA (B. Buss)

**Keywords:** Sporotrichosis, Sporothrix schenckii, fungi, zoonoses, domestic cats, veterinary, technician, Kansas, zoonotic disease, fungal infection, cat-transmitted sporotrichosis, animal health, One Health, occupational health

## Abstract

We describe a feline sporotrichosis cluster and zoonotic transmission between one of the affected cats and a technician at a veterinary clinic in Kansas, USA. Increased awareness of sporotrichosis and the potential for zoonotic transmission could help veterinary professionals manage feline cases and take precautions to prevent human acquisition.

Sporotrichosis, an implantation mycosis caused by fungi in the genus *Sporothrix*, affects humans and other mammals. Although cat-transmitted sporotrichosis caused by the highly transmissible *Sporothrix brasiliensis* species is an increasing concern in Latin America (*1*), *S. brasiliensis* has not been detected in the United States, and cat-transmitted *Sporothrix schenckii* is rarely reported (*2*,*3*). We describe a cluster of sporotrichosis cases involving 2 domestic cats and zoonotic transmission between one of the affected cats and a veterinary technician in Kansas, USA.

In August 2022, a pregnant, 2-year-old, indoor-outdoor cat was brought to a veterinary clinic in northwest Kansas with an ulcerated lesion on her distal paw thought to be from a cat fight. She was initially treated with amoxicillin-clavulanic acid, but the wound worsened over the next month and additional ulcerated lesions developed along the rest of the forelimb (Figure, panel A).

**Figure:**
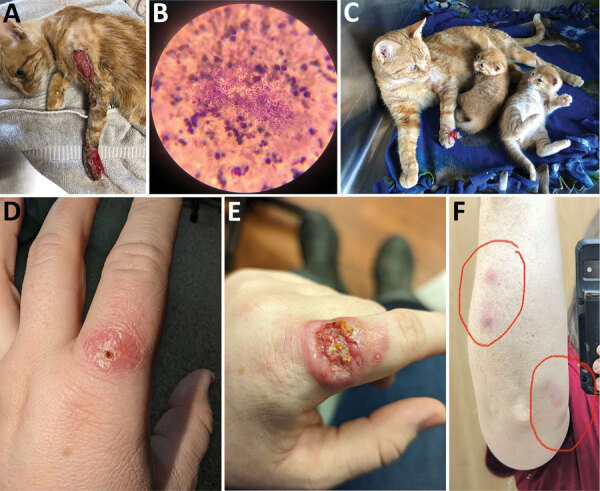
Clinical manifestations seen in pregnant 2-year-old cat and human from a sporotrichosis cluster in domestic cats and veterinary technician, Kansas, USA, 2022. A) Severe lesion on cat 1 in August 2022; B) cytological examination from cat 1’s lesions showing numerous cigar-shaped yeasts consistent with *Sporothrix*; C) image of cat 1 with kittens and improved lesion in September 2022; D) lesion on the finger of a veterinary technician who had contact with cat 1; E) ulcerated and more severe lesion on technician’s finger after X days; F) lymphadenopathy on technician’s arm. Cat 1 initially was treated with antibiotics in August 2022 and lesions improved on antifungal therapy. However, the cat’s lesions returned and worsened in October 2022, after discharge from the facility. In November 2022, a veterinary technician developed a small lesion 1 week after being poked through the glove by a claw on cat 1’s infected paw. Lymphadenopathy progressed up the technician’s arm (red circles, panel F) in a sporotrichoid pattern along dermal and lymphatic vessels.

The veterinarian performed an impression smear, which revealed cytology consistent with *Sporothrix* (Figure, panel B). The cat was treated with 10 mg/kg itraconazole and meloxicam in addition to amoxicillin-clavulanic acid. The cat improved on antifungal medication and gave birth to 2 healthy kittens in September 2022 (Figure, panel C). However, lesions reappeared after 1 month and began to extend up the limb. Treatment was adjusted to include terbinafine at 30 mg/kg, but the lesions continued to worsen and spread to the other 3 limbs. The cat was humanely euthanized, and the remains were cremated.

In November 2022, a veterinary technician caring for cat 1 at the clinic received a puncture wound through the glove from the cat’s infected paw. A small blister developed at the puncture site approximately 2 weeks after the scratch (Figure, panel D). The blister quickly ulcerated, and the technician developed sporotrichoid lymphadenopathy up her arm (Figure, panels E, F). The technician began a treatment of cephalexin and then switched to 200 mg oral itraconazole twice daily and doxycycline. Cultures completed at Nebraska Medical Center were positive for *Sporothrix* spp., and an isolate was sent to the US Centers for Disease Control and Prevention (CDC) for identification. The isolate was identified as *S. schenckii* based on Sanger sequencing of the calmodulin gene (Appendix). Whole-genome sequencing showed the isolate clustered with historical *S. schenckii* isolates from the United States (bootstrap value of 100%) (Appendix Figure). The technician completed 8 months of oral itraconazole and recovered.

In February 2023, cat 1’s owners brought another indoor-outdoor cat from the same property to the veterinary clinic with similar lesions. Cytology revealed *Sporothrix*. Cat 2 underwent a 4-month regimen of itraconazole 10 mg/kg, and the lesions healed.

This report describes a cluster of feline sporotrichosis cases in 2 indoor-outdoor cats and zoonotic transmission between 1 of the cats and a veterinary technician. The disease course of cat 1 highlights the potential severity of feline sporotrichosis. Early diagnosis of sporotrichosis and early treatment initiation with appropriate antifungal drugs can improve outcomes and help prevent transmission to other cats or humans (*4*). Cytology and culture should be considered for wounds or lesions that fail to respond to antibiotics. Itraconazole should be given to cats with food to improve absorption, and potassium iodide in combination with itraconazole can improve treatment efficacy in cats with multiple or extensive lesions or in treatment refractory cases (*5*,*6*).

This sporotrichosis cluster raised concerns that *S. brasiliensis* could be the etiologic agent. *S. brasiliensis* has increasingly been reported in Latin America (*1*), and 3 cases were recently reported in the United Kingdom, highlighting the potential for international spread (*7*). However, the etiologic agent in our report was *S. schenckii*, which is typically acquired through traumatic contact with plant matter. Although rare, cat-transmitted *S. schenckii* cases have been reported in the United States and Southeast Asia (*2*,*3*,*8*).

To reduce zoonotic transmission risk, veterinary professionals should wear examination gloves when handling cats with suspected sporotrichosis and take precautions to avoid scratches or bites (*9*). Wounds from scratches or bites should be washed promptly with soap and water (https://www.cdc.gov/healthypets/pets/cats.html). Persons who have close contact with a cat with sporotrichosis should seek healthcare promptly if they develop lesions or sporotrichoid lymphadenopathy (*1*,*3*).

This report was limited by a lack of detailed exposure information for how the cats acquired sporotrichosis. Nevertheless, keeping cats indoors is recommended to prevent environmental acquisition of sporotrichosis (*1*,*2*). Risk factors for feline acquisition of sporotrichosis likely resemble those for humans, including traumatic inoculation or wound contamination with hay, roses, or sphagnum moss, or bites or scratches from other cats (*9*,*10*). Intact male, free-roaming cats might be at increased risk for sporotrichosis (*10*). Cats with sporotrichosis should be kept indoors and apart from other cats in the home to reduce the potential for further transmission. In conclusion, increased awareness of sporotrichosis in cats and the potential for zoonotic transmission could help veterinary professionals more quickly recognize and treat feline cases and take precautions to prevent human acquisition in the veterinary setting.

Appendix Additional information on sporotrichosis cluster in domestic cats and veterinary technician, Kansas, USA, 2022.
