# Supplementary material for: Sporotrichosis Cluster in Domestic Cats and Veterinary Technician, Kansas, USA, 2022
Source: Emerg Infect Dis. 2024 May;30(5):1053–5. doi: 10.3201/eid3005.231563 (PMC11060436; doi:10.3201/eid3005.231563)
Supplement: Appendix — Additional information on sporotrichosis cluster in domestic cats and veterinary technician, Kansas, USA, 2022. [file 23-1563-Techapp-s1.pdf]

Article DOI: https://doi.org/10.3201/eid3005.231563

EID cannot ensure accessibility for supplementary materials supplied by authors. Readers who have difficulty accessing supplementary content should contact the authors for assistance.

Sporotrichosis Cluster in Domestic Cats and Veterinary Technician, Kansas, USA, 2022

Appendix

Species Identification

Species identification was conducted at the Mycotic Diseases Branch Laboratory at CDC, Atlanta, USA. DNA was extracted using the DNeasy Blood and Tissue kit (Qiagen, Gaithersburg, MD, USA) according to the manufacturer's instructions. Quantity of the DNA was measured by a Nanodrop 2000c at a wavelength of 260A (Thermo Fisher Scientific, Pittsburgh, PA). PCR sequencing, purification of PCR products and Sanger sequencing were performed as previously described in Gade et al. (*1*), including the PCR conditions and purification of the PCR products by ExoSAP (Affymetrix, Santa Clara, CA) according to the manufacturer's instructions. The calmodulin (CAL) locus region was amplified directly from the genomic DNA using primers CL1-GARTWCAAGGAGGCCTTCTC and CL2A-TTTTTGCATCATGAGTTGGAC, as described in O'Donnell et al. (*2*) and Rodrigues et al. (*3*) for species confirmation.

Genomic Sequencing

Next, genomic libraries were constructed using NEBNext Ultra DNA Library Prep kit (New England Biolabs, Ipswich, MA, U.S.) for Illumina and sequenced on Illumina NovaSeq 6000SP reagent kit (500 cycles). Read data has been deposited into the SRA database (BioProject PRJNA1021525). An additional ten *S. schenckii* isolates from the United States and NCBI SRA (Appendix Table) were included in the genomic analysis for comparison to the isolate. SNPs were identified using MycoSNP v1.4 (https://github.com/CDCgov/mycosnp-nf/) as described by Bagal et al. (*4*). Analyses were conducted using *S. schenckii* strain 1099–18

reference genome (NCBI: txid 1397361) (*5*). A maximum likelihood (ML) tree from the filtered SNPs calling file was built using FastTree v2.1.11. Genetic distance calculations and neighbor-joining tree construction were performed using MEGA 11. The consensus topology, branch support, and maps were visualized using Microreact (http://microreact.org).

References

1. Gade L, Scheel CM, Pham CD, Lindsley MD, Iqbal N, Cleveland AA, et al. Detection of fungal DNA in human body fluids and tissues during a multistate outbreak of fungal meningitis and other infections. Eukaryot Cell. 2013;12:677–83. PubMed https://doi.org/10.1128/EC.00046-13

2. O'Donnell K, Nirenberg HI, Aoki T, Cigelnik E. A Multigene phylogeny of the *Gibberella fujikuroi* species complex: detection of additional phylogenetically distinct species. Mycoscience. 2000;41:61–78. https://doi.org/10.1007/BF02464387

3. Rodrigues AM, de Hoog S, de Camargo ZP. Emergence of pathogenicity in the *Sporothrix schenckii* complex. Med Mycol. 2013;51:405–12. PubMed https://doi.org/10.3109/13693786.2012.719648

4. Bagal UR, Phan J, Welsh RM, Misas E, Wagner D, Gade L, et al. MycoSNP: a portable workflow for performing whole-genome sequencing analysis of *Candida auris*. In: Lorenz A, editor. Candida auris. Methods in Molecular Biology, volume 2517. New York, NY: Springer US; 2022. p. 215–28 [cited 2023 Sep 26]. https://link.springer.com/10.1007/978-1-0716-2417-3_17

5. Teixeira MM, de Almeida LG, Kubitschek-Barreira P, Alves FL, Kioshima ÉS, Abadio AK, et al. Comparative genomics of the major fungal agents of human and animal Sporotrichosis: *Sporothrix schenckii* and *Sporothrix brasiliensis*. BMC Genomics. 2014;15:943. PubMed https://doi.org/10.1186/1471-2164-15-943

Appendix Table. Characteristics of whole-genome sequences downloaded from NCBI (https://www.ncbi.nlm.nih.gov/sra/?term = Sporothrix).

Sample accession no.	Strain	Year of isolation	Geographic origin	Host
SRR12483721	*Sporothrix schenckii* SsEM7	2012	Colombia	*Homo sapiens*
SRR12483722	*Sporothrix schenckii* SsMS1	2012	Colombia	*Homo sapiens*
SRR12483724	*Sporothrix schenckii* A0003	2015	Brazil	*Felis catus*
SRR24215198	B22064	2021	Brazil	*Homo sapiens*
SRR24215187	B22065	Unknown	Brazil	*Homo sapiens*
SRR2421519	B22147	2022	Brazil	*Homo sapiens*
SRR24215210	B22103	2016	Brazil	*Felis catus*
SRR24215243	B10282	2013	United States	*Homo sapiens*
SRR24215242	B11252	2015	United States	*Homo sapiens*
SRR26324850	B24667	2022	United States	*Felis catus*

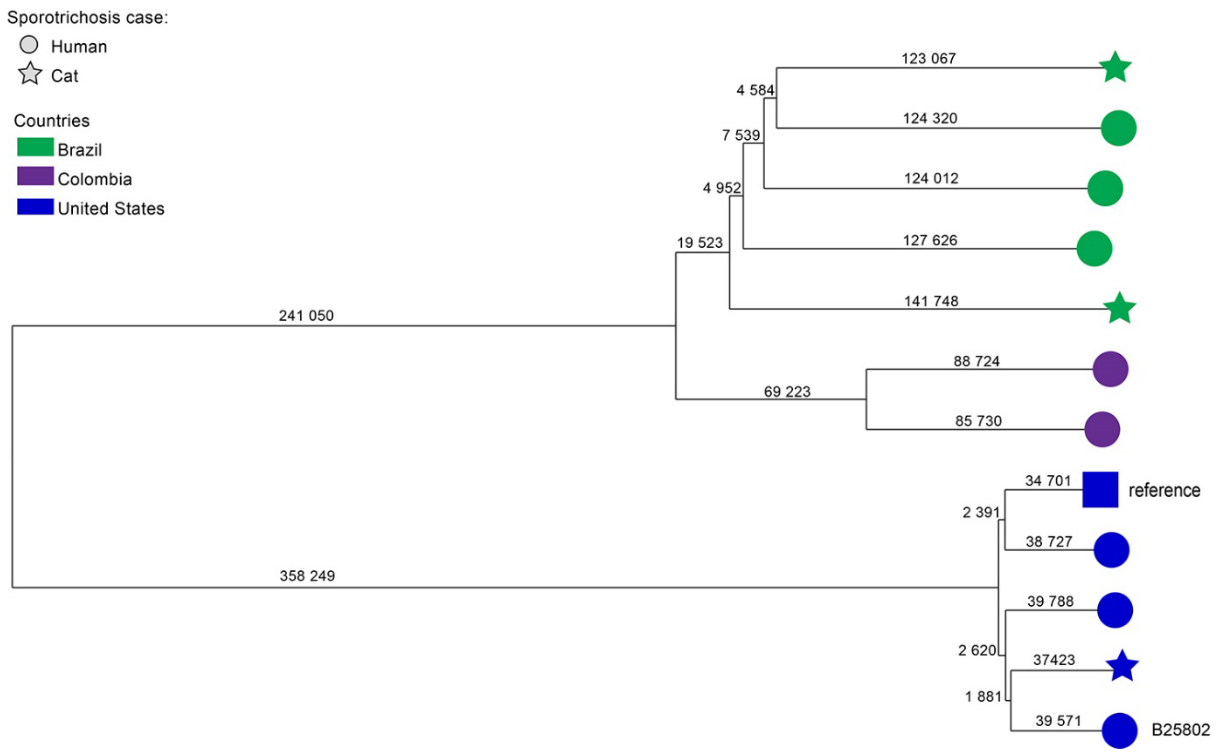

Appendix Figure. Neighbor-joining phylogenetic tree of *Sporothrix schenckii* isolates from Brazil, Colombia, and the United States. Shapes represent the *S. schenckii* host (human or cat); the square represents the S. schenckii strain 1099–18 reference genome (NCBI: 1397361). Branch lengths represent SNPs. Isolate from human case in Kansas is identified (B25802).
